# Long-term outcomes of the “pave-and-crack” technique for severely calcified femoropopliteal chronic total occlusions

**DOI:** 10.1186/s42155-026-00766-9

**Published:** 2026-09-04

**Authors:** Birte Winther, Andrej Schmidt, Lena Rummler, Axel Fischer, Sandra Düsing, Sabine Steiner, Xiao Di, Tobias Hirsch, Dierk Scheinert, Tim Wittig

**Affiliations:** 1 Division of Angiology, Department of Internal Medicine, Neurology and Dermatology, University Hospital Leipzig, Leipzig, Germany; 2 Helmholtz Institute for Metabolic, Obesity and Vascular Research (HI-MAG) of the Helmholtz Center Munich at the University of Leipzig and University Hospital Leipzig, Leipzig, Germany; 3 Division of Angiology, Department of Medicine II, Medical University Vienna, Vienna, Austria; 4 Department of Vascular Surgery, Peking Union Medical College Hospital, Chinese Academy of Medical Sciences and Peking Union Medical College, Beijing, China; 5 Practice for Internal Medicine and Vascular Diseases, Halle, Germany

**Keywords:** Peripheral artery disease, Femoropopliteal segment, Chronic total occlusion, Endovascular therapy

## Abstract

**Objective:**

To evaluate the long-term safety and efficacy of the pave-and-crack technique, combining aggressive balloon dilatation and covered stent implantation, for endovascular therapy (EVT) of highly calcified femoropopliteal chronic total occlusions (CTOs) in poor surgical candidates unsuitable for open revascularization or conventional EVT.

**Design:**

Single-center retrospective cohort study based on a prospectively maintained peripheral artery disease database.

**Methods:**

Patients with symptomatic femoropopliteal CTOs treated using the pave-and-crack technique between September 2014 and April 2025 were included. Lesion and procedural characteristics, technical success, defined as residual stenosis ≤ 30%, complications, major target limb amputation, and symptom status were assessed. Kaplan–Meier analysis was used to estimate freedom from target lesion revascularization (TLR) and all-cause mortality through 5 years.

**Results:**

A total of 122 patients with 136 femoropopliteal CTOs were treated. Mean lesion length was 253.0 ± 90.1 mm, and 93.4% (127/136) of lesions were severely calcified. Retrograde access was required in 72.8% (99/136) of procedures. Technical success was achieved in 93.4% (127/136). Freedom from target lesion revascularization was 91.4% ± 2.6% at 1 year, 77.9% ± 4.6% at 3 years, and 66.7% ± 7.2% at 5 years. Major target limb amputation occurred in four cases presenting with CLTI (8.5%; 4/47) and none with claudication (0%; 0/89). Five-year freedom from all-cause mortality was 71.4 ± 6.9% for claudicants and 41.4 ± 11.2% for patients with CLTI (*p* < 0.01).

**Conclusion:**

The pave-and-crack technique is feasible for highly calcified femoropopliteal CTOs, achieving high technical success, encouraging long-term freedom from TLR, and high limb salvage.

**Graphical Abstract:**

Long-Term Outcomes of the “Pave-and-Crack” Technique for Severely Calcified Femoropopliteal Chronic Total Occlusions. (Left) Lesion and Procedural Characteristics. (Middle) the “Pave-and-Crack” Technique (A) Fluoroscopy showing aggressive balloon pre-dilatation. (B) Angiography showing vessel rupture. (C) Fluoroscopy following covered stent implantation (Viabahn) and relining with an interwoven nitinol stent (Supera). (D) Final angiography. (Right) Kaplan Meier survival curves and estimates for freedom from target lesion revascularization over five years stratified for popliteal involvement (segment P1-P3). CLTI: Chronic limb threatening ischemia. CTO: Chronic total occlusion. PACSS: Peripheral Calcification Scoring System. PTA: Percutaneous transluminal angioplasty. KM: Kaplan-Meier, SD: Standard deviation, TLR: Target lesion revascularization.

**Figure:**
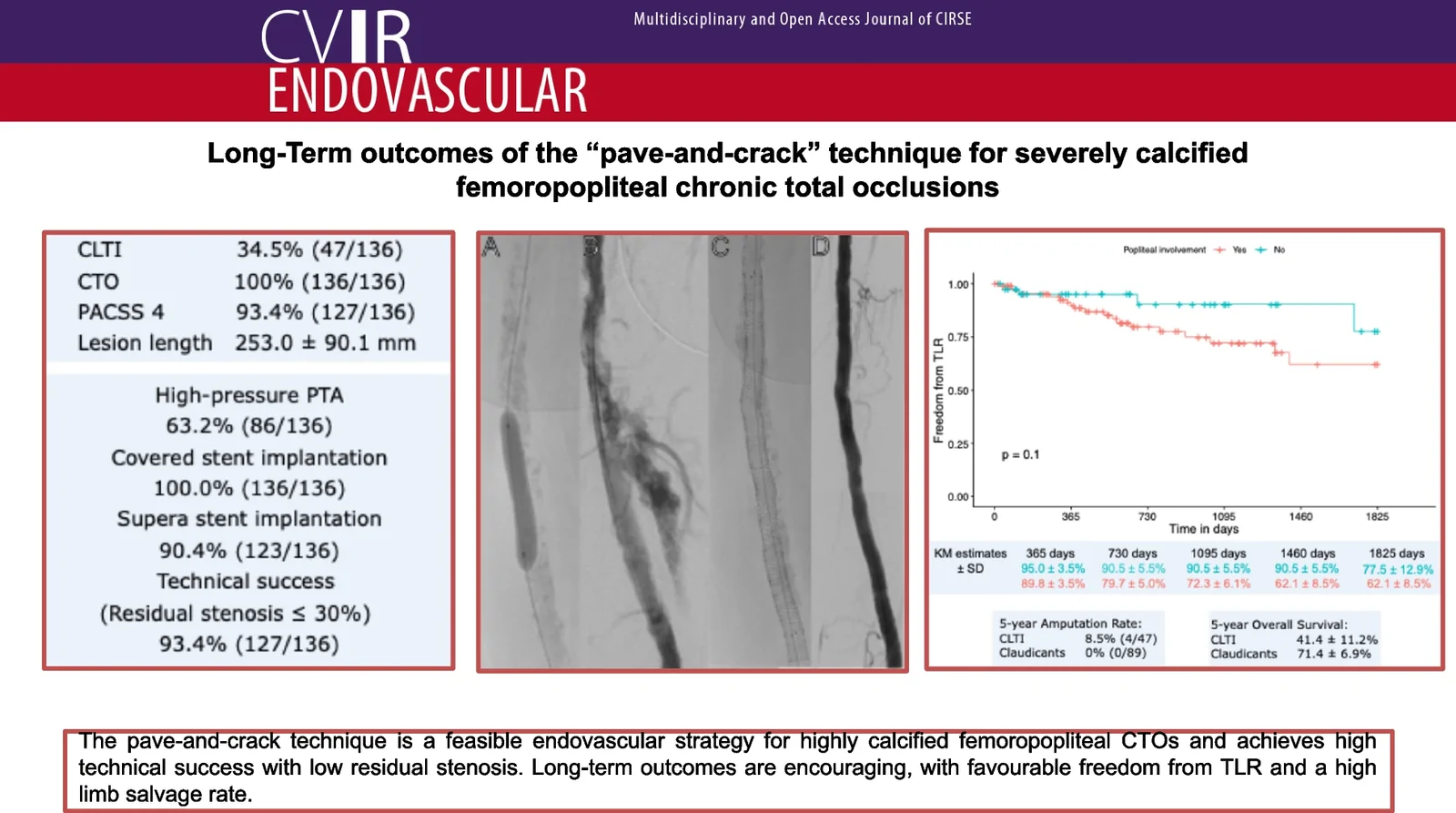

**Supplementary Information:**

The online version contains supplementary material available at https://doi.org/10.1186/s42155-026-00766-9.

## Introduction

With an aging population and the rising prevalence of diabetes mellitus and chronic kidney disease, peripheral artery disease (PAD) is increasingly characterized by long chronic total occlusions (CTOs) and severe arterial calcification [1]. These lesions often occur in patients with substantial comorbidities and elevated perioperative risk, rendering many unsuitable candidates for open surgical revascularization. Consequently, endovascular therapy (EVT) has become the preferred first-line revascularization strategy in many patients with complex femoropopliteal occlusive disease [2]. However, EVT remains technically challenging and may fail in up to 20% of complex CTOs, primarily because of severe calcification, difficult lesion crossing, and unsuccessful reentry [3, 4].

Beyond technical failure, severe calcification is associated with inferior durability after EVT. Calcified plaque may impair drug uptake after drug-coated balloon (DCB) angioplasty, promote elastic recoil and flow-limiting dissection after percutaneous transluminal angioplasty (PTA), and increase the need for bailout stenting due to residual stenosis [5–7]. These limitations are clinically relevant, as heavy calcification has been associated with higher rates of restenosis and limb loss [8, 9].

The pave-and-crack technique was introduced as an endovascular strategy for severely calcified femoropopliteal lesions in which conventional EVT is associated with insufficient lumen gain or an increased risk of vessel perforation, due to high-pressure balloon angioplasty required to address extensive vessel wall calcification. It combines aggressive balloon pre-dilatation to fracture calcified plaque with covered stent implantation to seal or prevent vessel injury, followed, when appropriate, by relining with an interwoven nitinol stent to provide additional radial support and minimize recoil. Previously published 1-year results demonstrated high technical success and encouraging outcomes regarding patency and limb salvage in long CTOs [10]. The aim of the present analysis was to evaluate the long-term clinical outcomes of the pave-and-crack technique in patients with heavily calcified femoropopliteal occlusive disease who were considered poor candidates for open revascularization.

## Material and methods

This single-center, retrospective analysis included patients with femoropopliteal CTOs who underwent EVT of symptomatic femoropopliteal lesions (Rutherford category 2–6) utilizing the pave-and-crack technique. The study protocol allowed for the inclusion of one femoropopliteal CTO per leg per patient, with no upper lesion length limit.

All patients provided written informed consent for data collection within a prospectively maintained PAD database. The study protocol was approved by the Institutional Review Board (EK Vote 223/23-ek) and was conducted in accordance with the ethical principles outlined in the Declaration of Helsinki.

### Procedure

Detailed patient characteristics were obtained from the electronic medical record, which is collected using standardized protocols at the time of admission. Lesion-specific and procedural details were extracted from the interventional report and review of angiograms. Lesion calcification was graded using the Peripheral Arterial Calcium Scoring System (PACSS) and plaque diameter assessed and measured on fluoroscopy [7].

EVT was routinely conducted under local anesthesia using a standardized contralateral crossover approach through the common femoral artery. Ipsilateral antegrade femoral access was used when clinically appropriate. If antegrade crossing of the lesion was unsuccessful because of lesion complexity, distal retrograde access was utilized. After vascular access had been established, 5000 IU of intravenous heparin were administered, followed by diagnostic angiography of the target limb. Due to anticipated pain during the procedure, local anesthesia was routinely administered either percutaneously or using a reentry device [11].

**Fig. 1:**
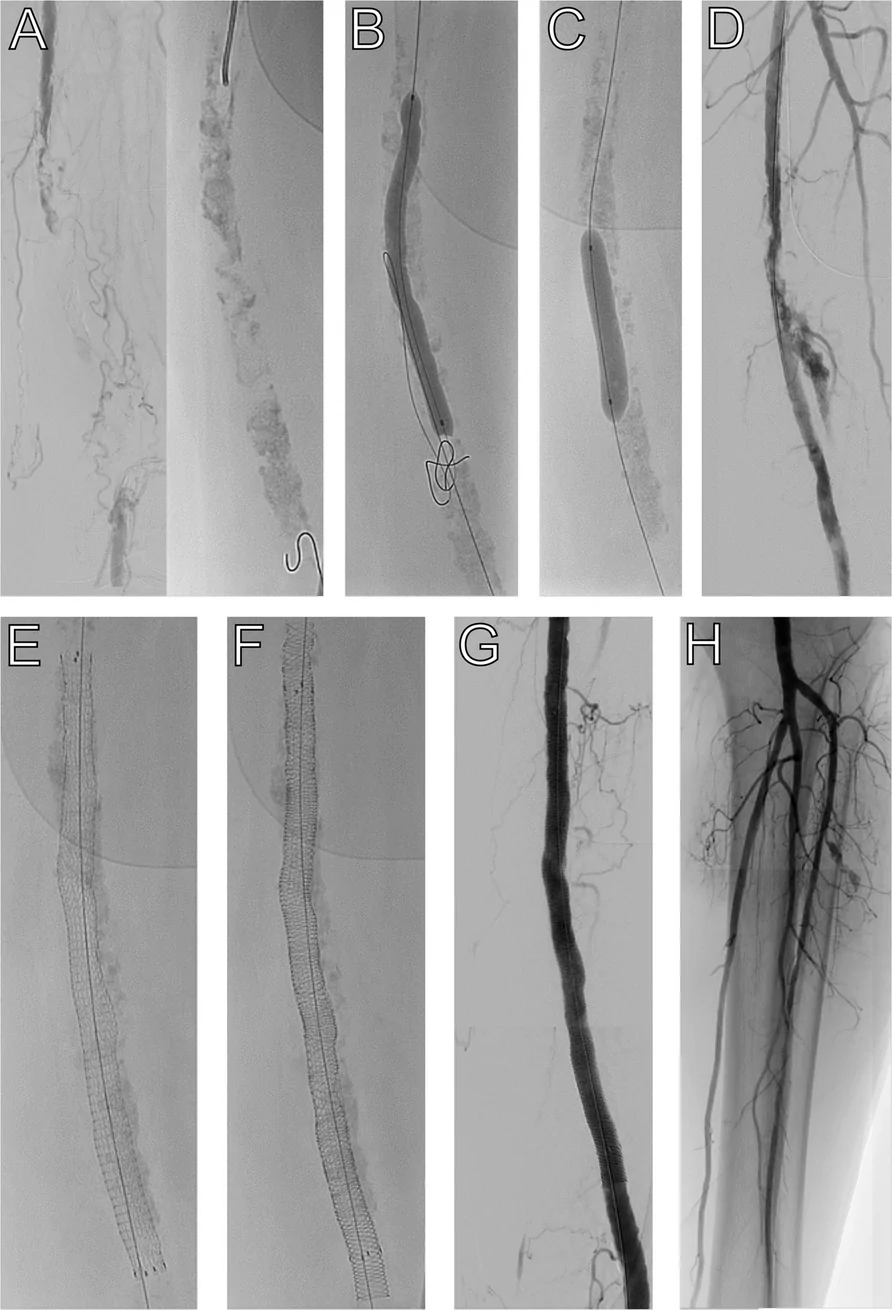
Case presentation of the pave-and-crack technique in a severely calcified occlusion of the left medio-distal superficial femoral artery (SFA). The case illustrates a 67-year-old male patient with symptomatic chronic total occlusion of the medio-distal SFA (Rutherford category 3). **A** Baseline angiography and fluoroscopy demonstrate extensive calcification and occlusion of the medio-distal SFA. **B** Lesion crossing was challenging and required combined antegrade and retrograde access via the proximal anterior tibial artery, with successful crossing ultimately achieved using the reversed Controlled Antegrade and Retrograde Tracking (CART) maneuver. **C** Aggressive balloon pre-dilatation was performed using balloons with increasing diameter (Sterling Balloon, 6/80, 7/40 mm; Boston Scientific, Marlborough, MA, USA), followed by high-pressure balloon angioplasty with Conquest balloons (07/20, 8/40 mm; Becton Dickinson, Franklin Lakes, NJ, USA). **D** High-pressure angioplasty resulted in a small perforation of the distal SFA. **E** The lesion was subsequently “paved” with implantation of an 8/150 mm Viabahn stent graft. **F** Relining was performed using Supera nitinol stents (7/100, 7/80, 7/40 mm). **G** Final angiography demonstrated an excellent postinterventional result. **H** Angiography of the outflow vessels with three-vessel runoff

The pave-and-crack technique has been described in detail previously (Fig. 1) [10]. In short, after crossing the lesion, thorough pre-dilation is performed with inflation times of at least 3 min and balloons at least matching the reference vessel diameter (RVD), often with deliberate oversizing to overcome recoil. RVD was measured using the diameter of the balloon inflated within the lesion compared to the non-diseased segment using roadmap. If full balloon expansion is not achieved, focal or high-pressure dilation is performed using short balloons, scoring balloons, noncompliant balloons, or larger high-pressure balloons until the calcified plaque is “cracked.” Due to severe perforation, or anticipated rupture because of aggressive pre-dilatation, a Viabahn stent-graft (W. L. Gore & Associates, Newark, DE, USA) is implanted to “pave” the treated segment and seal or prevent vessel injury (crack-and-pave versus pave-and-crack) in all cases. Finally, the Viabahn stent graft can be relined with appropriately sized Supera stents (Abbott Laboratories, Abbott Park, North Chicago, IL, USA), usually 0.5 mm smaller in diameter than the covered stent, to provide additional radial support and avoid persistent recoil in heavily calcified segments. In less calcified lesion segments, DCBs and drug-eluting stents (DES) were used for additional treatment proximal or distal to the Viabahn stent graft.

### Medication

Following covered stent implantation, patients received dual antiplatelet therapy or, in those requiring oral anticoagulation, oral anticoagulation combined with an additional antiplatelet agent for at least 6 months. Thereafter, long-term single-agent therapy was continued with aspirin (100 mg/day), clopidogrel (75 mg/day), or oral anticoagulation, as clinically indicated. In patients who were not receiving antiplatelet therapy before the procedure, a loading dose of either aspirin (500 mg) or clopidogrel (300 mg) was administered prior to the intervention.

### Endpoints and follow-up

The efficacy endpoint was 5-year freedom from target lesion revascularization (TLR), defined as reintervention for angiographically confirmed diameter stenosis ≥ 50% within 5 mm of the target lesion. The safety endpoint was freedom from procedure-related death through 30 days, major target limb amputation, and overall survival over the 5-year follow-up period. Technical success was defined as a final in-lesion residual diameter stenosis ≤ 30%, as assessed by the operator [12].

Follow-up was performed at 6 months and annually thereafter, either during in-house visits or by the treating outpatient physician. Follow-up assessments included the need for TLR, major target limb amputation, symptom status according to Rutherford category, and mortality. If no follow-up data were available from clinical records, patients were contacted by telephone to obtain information on these outcomes. Patients were considered lost to follow-up if two telephone contact attempts and subsequent contact with the primary physician were unsuccessful.

### Statistical analysis

Due to the retrospective nature of the study, no formal sample size calculation was conducted. Kaplan–Meier (KM) survival analysis was used to evaluate freedom from TLR and all-cause mortality through 1825 days (5 years). Significance in stratified KM analyses was assessed using the log-rank test. A two-tailed *p* value of < 0.05 was considered statistically significant. All statistical analyses were performed using R version 2026.04.0 + 526 (Posit Software, PBC, Boston, MA, USA).

## Results

Between September 2014 and April 2025, 122 patients with 136 femoropopliteal CTOs underwent EVT utilizing the pave-and-crack technique. Twenty-seven patients undergoing 28 interventions between December 2014 and June 2016 were included in the previous publication reporting 12-month outcomes of the pave-and-crack technique [10]. Cardiovascular risk factors were prevalent in this predominantly male cohort (83.6%; 102/122), with hypertension and hyperlipidemia present in 95.1% (116/122), and diabetes mellitus in 53.3% (65/122) of patients. In one-third (34.6%; 47/136) of cases, patients presented with chronic limb-threatening ischemia (CLTI) at baseline, defined by the presence of ischemic rest pain and/or tissue loss. Patient characteristics are detailed in Table 1.

**Table 1:** Patient demographics and medical history

Demographics	
Age (years)	71.4 ± 8.5
Male sex	102 (83.6)
Diabetes mellitus	65 (53.3)
Hypertension	116 (95.1)
Hyperlipidemia	116 (95.1)
Coronary artery disease	75 (61.5)
Cerebrovascular disease	32 (26.2)
Heart insufficiency	29 (23.8)
Renal insufficiency^†^	68 (55.7)
Smoking history	
Never	23 (18.9)
Former	64 (52.5)
Current	35 (28.7)
Previous surgeries^*^	
TEA	43 (31.6)
Bypass	22 (16.2)
Clinical presentation^‡,*^	
2	18 (13.2)
3	71 (52.2)
4	7 (5.1)
5	36 (26.5)
6	4 (2.9)
Baseline medication	
ASS	94 (77.0)
Clopidogrel	37 (30.3)
Direct factor Xa inhibitors	25 (20.5)
Vitamin K antagonist	4 (3.3)
Heparin	7 (5.7)
Statin	105 (86.1)
Another lipid-lowering agent	32 (26.2)

*N* = 122. Data are expressed as mean ± SD for continuous variables or as *n* (%) for categorial variables. *TEA* thromboendarterectomy

^†^Defined as glomerular filtration rate < 60 ml/min/1.73 m^2^

^*^Reported per limb, *N* = 136

^‡^Classified according to the Rutherford classification

Lesion characteristics are summarized in Table 2. Mean lesion length was 253.0 ± 90.1 mm. According to the PACSS classification, CTOs were heavily calcified, defined as PACSS grade 4, in 93.4% of cases (127/136). Most lesions were de novo lesions (61.0%; 83/136), whereas re-stenotic lesions accounted for 24.3% (33/136) and in-stent restenosis for 14.7% (20/136).

**Table 2:** Lesion characteristics

Lesion characteristics	
Vessel segment^*^	
SFA	129 (94.9)
P1	88 (64.7)
P2	64 (47.1)
P3	27 (19.9)
BTK	3 (2.2)
Lesion type	
De novo	83 (61.0)
Re-stenotic	33 (24.3)
ISR	20 (14.7)
CTO	136 (100.0)
Lesion length (mm)	253.0 ± 90.1
Treated length (mm)	261.4 ± 92.4
Severe calcification^†^	127 (93.4)
Plaque diameter^‡^ (mm)	6.1 ± 0.9

*N* = 136. Data are expressed as mean ± SD for continuous variables or as *n* (%) for categorial variables. *SFA* superficial femoral artery, *P1* popliteal artery, segment 1, *P2* popliteal artery, segment 2, *P3* popliteal artery, segment 3, *BTK* below the knee, *ISR* in-stent restenosis, *CTO* chronic total occlusion

^*^Multiple answers possible

^†^According to the Peripheral Arterial Calcification Scoring System (PACSS 4)

^‡^As assessed on fluoroscopy

Procedural characteristics are presented in Table 3. Retrograde access following unsuccessful or impossible antegrade guidewire passage was required in 72.8% of cases (99/136), and a reentry device was used in 36.8% of procedures (50/136). In all cases, initial PTA resulted in suboptimal outcomes with high residual stenosis, necessitating further treatment. Additional vessel preparation was considered unlikely to be successful because of severe calcification and further PTA deemed to carry a high risk of vessel rupture. Approximately half of the lesions were treated using the crack-and-pave technique, with aggressive PTA followed by covered stent implantation, whereas the remaining lesions were treated using the pave-and-crack technique, in which covered stent placement precedes aggressive PTA to mitigate the risk of major bleeding following vessel rupture. Between 2014 and 2020, 34.7% of interventions (25/72) were performed using the pave-and-crack technique and 65.3% (47/72) using the crack-and-pave technique. Between 2021 and 2025, this distribution shifted, with 71.9% interventions (46/64) performed using the pave-and-crack technique and 28.1% (18/64) using the crack-and-pave technique. On average, 1.4 ± 0.6 Viabahn stent grafts were implanted per lesion, with a mean diameter of 5.8 ± 0.7 mm. In almost half of the cases (45.6%; 62/136), the Viabahn diameter was 7 mm or larger. In 90.4% of cases (123/136), relining with an interwoven nitinol stent was performed, with a mean stent diameter of 5.8 ± 0.6 mm.

**Table 3:** Procedural characteristics and periprocedural complications

Procedural characteristics	
Type of intervention	
Pave and crack	71 (52.2)
Crack and pave	65 (47.8)
Viabahn stent graft	136 (100.0)
Number of devices	1.43 ± 0.64
Maximal diameter (mm)	6.60 ± 0.66
Cumulative length (mm)	222.50 ± 112.54
Supera stent	123 (90.4)
Number of devices	1.76 ± 0.68
Maximal diameter (mm)	5.84 ± 0.56
Cumulative length (mm)	226.83 ± 113.57
Other treatment modalities^*^	134 (98.5)
High-pressure PTA	86 (63.2)
Scoring PTA	7 (5.1)
Cutting PTA	2 (1.5)
DCB PTA	67 (49.3)
BMS	38 (27.9)
DES	35 (25.7)
POBA	136 (100.0)
Atherectomy	17 (12.5)
Reentry device	50 (36.8)
Beback	43 (31.6)
Pioneer	3 (2.2)
Retrograde access	99 (72.8)
Inflow treated	11 (8.1)
Outflow treated	28 (20.6)
Technical success^†^	127 (93.4)
Complications	
Access-site complication	5 (3.7)
PSA	3 (2.2)
AV-fistula	1 (0.7)
Bleeding, stent implantation	1 (0.7)
Distal embolization	6 (4.4)

*N* = 136. Data are expressed as mean ± SD for continuous variables or as *n* (%) for categorial variables. *PTA* percutaneous balloon angioplasty, *DCB* drug-coated balloon, *BMS* bare metal stent, *DES* drug-eluting stent, *POBA* plain old balloon angioplasty, *PSA* pseudoaneurysm, *AV* arteriovenous

^*^Multiple answers possible

^†^Defined as final in-lesion residual diameter stenosis ≤ 30%

After plain old balloon angioplasty (POBA) (100.0%, 136/136), high-pressure balloons were used most frequently (64.2%; 86/136), followed by DCB angioplasty proximal and/or distal to the Viabahn stent graft (49.3%; 67/136). Technical success was achieved in 93.4% of cases (127/136).

Procedural complications are presented in Table 3. Access-site complications occurred in 3.7% of cases (5/136), and distal embolization in 4.4% (6/136). One patient died within 30 days of the intervention due to bronchial carcinoma and no deaths were considered procedure-related.

### Long-term follow-up

**Fig. 2:**
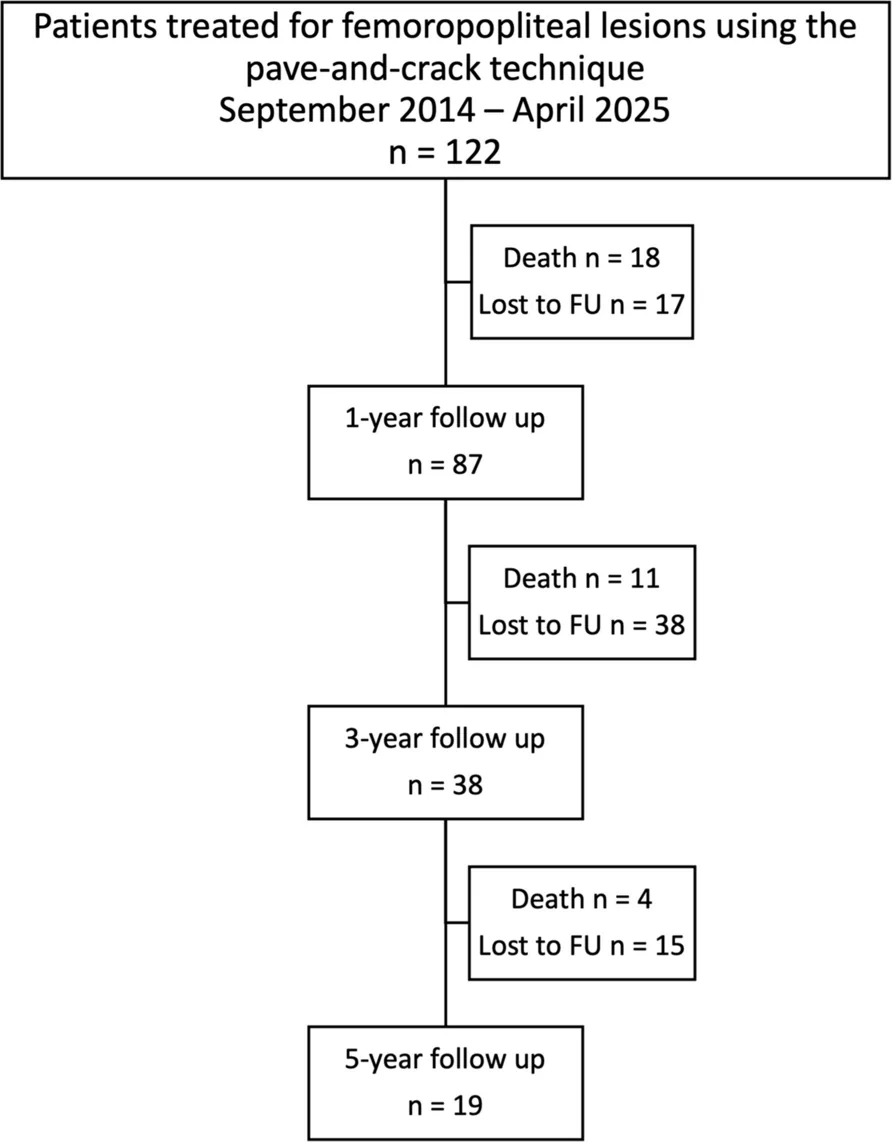
Flow diagram through 5-year follow-up. FU: follow-up

**Fig. 3:**
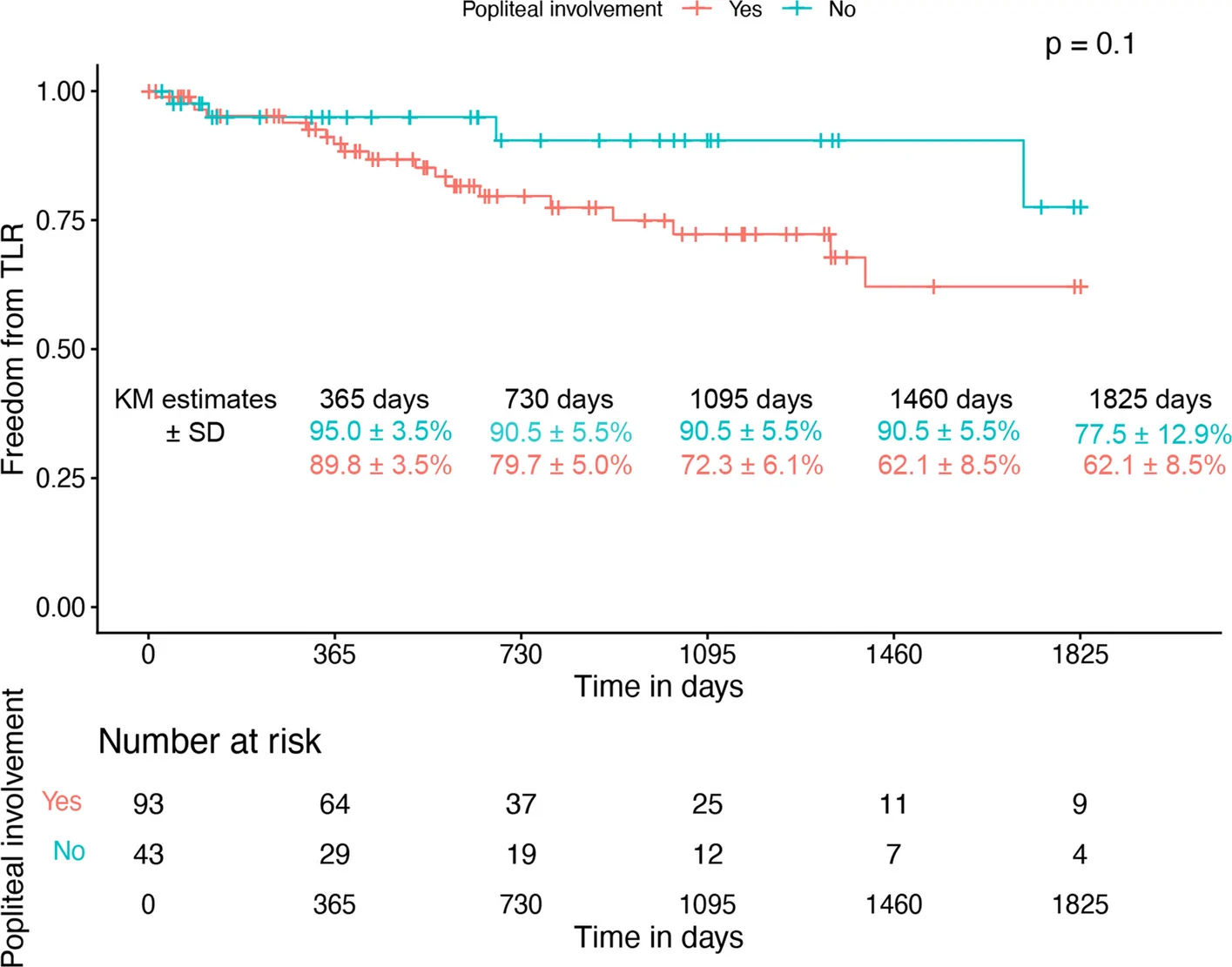
Freedom from target lesion revascularization stratified for popliteal involvement. Kaplan–Meier survival curves and estimates for freedom from target lesion revascularization over 5 years stratified for popliteal involvement (segment P1–P3) with corresponding numbers at risk. KM: Kaplan–Meier, SD: standard deviation, TLR: target lesion revascularization

**Fig. 4:**
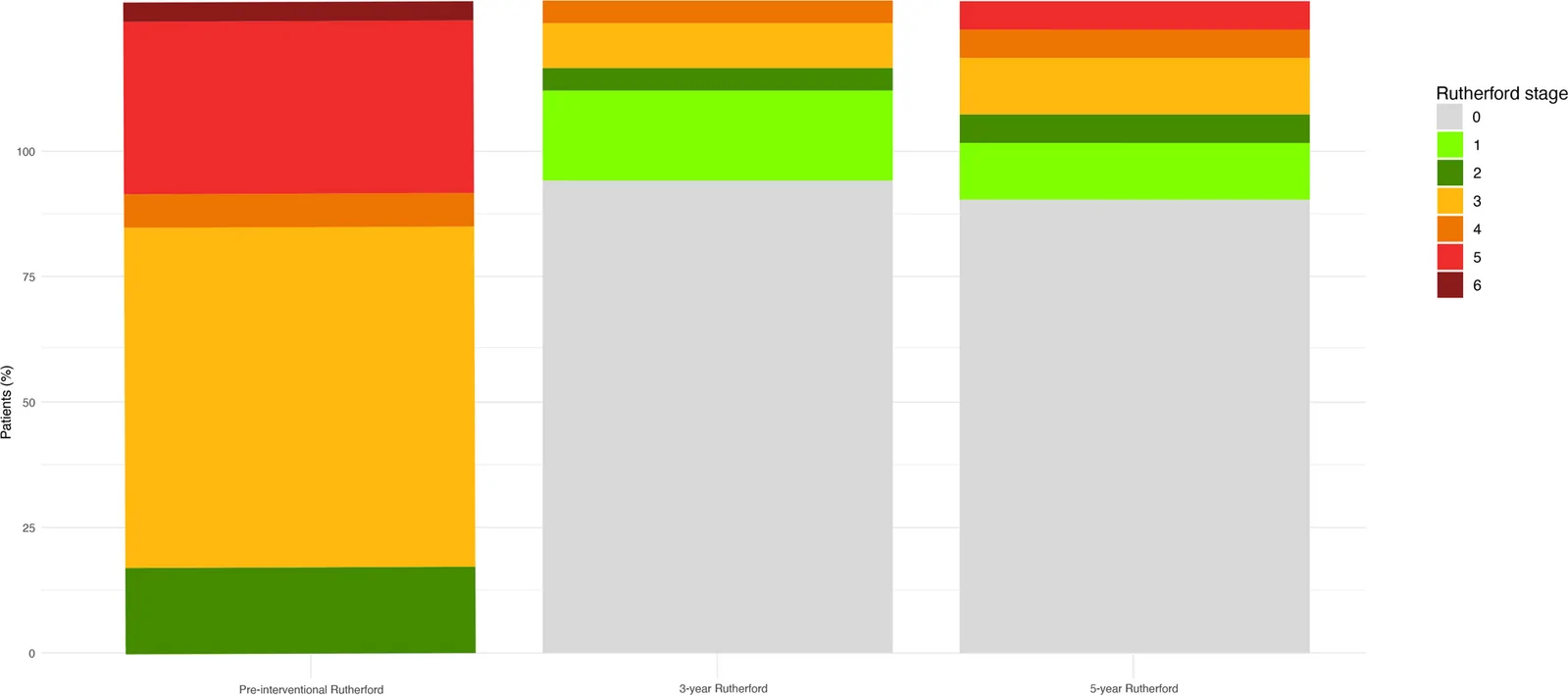
Rutherford stages pre-interventional and at 3-year follow-up. Rutherford stages are reported for all patients pre-interventional (*n* = 136) and for subset of patients at 3 years (*n* = 29) and 5 years (*n* = 23)

Mean follow-up was 1074 ± 861 days (Fig. 2). KM estimates of freedom from TLR were 91.4% ± 2.6% (95% confidence interval [CI] 86.4–96.7%) at 1 year, 83.0% ± 3.9% (95% CI 75.7–90.9%) at 2 years, 77.9% ± 4.6% (95% CI 69.3–87.5%) at 3 years, 70.8% ± 6.4% (95% CI 59.4–84.5%) at 4 years, and 66.7% ± 7.2% (95% CI 53.9–82.5%) at 5 years (Fig. S1). When stratified by lesion location, lesions with popliteal involvement showed a trend toward lower freedom from TLR compared with lesions confined to the femoral artery, although this trend did not reach statistical significance (log-rank *p* = 0.10; Fig. 3). At baseline, the mean Rutherford category was 3.52 ± 1.14, compared to 0.69 ± 1.26 at 3 years and 0.83 ± 1.50 at 5 years. Symptom status at 5-year follow-up, assessed according to Rutherford category, is shown in Fig. 4.

Five-year freedom from all-cause mortality was significantly higher among patients presenting with claudication than among those with CLTI, with KM estimates of 71.4 ± 6.9% (95% CI 59.0–86.3%) and 41.4 ± 11.2% (95% CI 24.3–70.4%), respectively (*p* < 0.01) (Fig. S2). Causes of death through 365 days of follow-up are presented in Table S1. At 5-year follow-up, major target limb amputation was reported in 4 patients presenting with CLTI (Rutherford category 6) at baseline. No patients presenting with claudication underwent major target limb amputation.

## Discussion

Severely calcified CTOs of the femoropopliteal segment remain a major challenge in contemporary EVT. Conventional recanalization techniques have been associated with suboptimal technical success and limited long-term outcomes, due to high residual stenosis rates, which has been associated with reduced efficacy [9, 13]. The pave-and-crack technique, originally developed to facilitate safe delivery of aortic stent grafts through diseased access vessels [14], has since been shown to be feasible for the treatment of femoropopliteal CTOs [10]. In the present study, we report the long-term safety and efficacy outcomes of the pave-and-crack technique in lesions that otherwise pose substantial technical challenges for EVT.

Overall, CTOs treated with the pave-and-crack technique were long and severely calcified. Retrograde access was required in more than 70% of cases, due to unsuccessful or unfeasible antegrade guidewire passage, underscoring the complexity of the lesions. Despite this challenging lesion morphology, procedural complications and the rate of residual stenosis remained low. Long-term mortality was high, particularly among patients presenting with CLTI, reflecting the clinical complexity and comorbidity burden of this population, which were associated with an elevated risk of general anesthesia and rendered many patients unsuitable for open surgical revascularization.

In our cohort, overall freedom from TLR at 5 years was approximately 60% and increased to nearly 80% in lesions confined to the femoral artery, while limb salvage was achieved in over 90% in patients with CLTI with durable symptom relief in claudicants. Overall, comparison of our long-term outcomes with the existing literature is challenging, as lesions of this complexity are rarely represented in published studies. The prospective, single-arm, multicenter DETOUR2 trial evaluated mid-term outcomes after percutaneous endovascular bypass for similarly complex femoropopliteal disease, including a mean lesion length of 330 mm, CTOs in 96% of cases, and severe calcification in 70%. In 202 patients, the study reported a technical success rate of 100% and a comparable 1-year freedom from TLR of nearly 90% [15]. However, long-term follow-up is ongoing, and future data will determine the durability and long-term efficacy of this technique.

In a prospective multicenter study evaluating long-term outcomes after Viabahn implantation in more than 300 complex femoropopliteal lesions with a mean lesion length of 24 cm, approximately three-quarters of patients remained free from TLR at 5 years, which is comparable to our findings [16]. However, only 40% of lesions in that study were moderately or severely calcified, and no lesion-crossing failures were reported, likely reflecting less complex lesion morphology overall. Another prospective multicenter study investigating Viabahn implantation predominantly in patients with claudication included less complex lesions (mean lesion length 22 cm; 66% CTO) and reported a 5-year freedom from TLR of 80% [17]. Importantly, that study included only lesions without popliteal involvement, and severe calcification was present in only 1% of cases. Against this background, the outcomes observed in our cohort appear comparable despite substantially more complex lesion characteristics. By contrast, a study evaluating nitinol stent implantation without drug-eluting technology, atherectomy, or scoring devices in long, highly calcified lesions, 80% of which were CTOs, reported comparable freedom from TLR of 60% at 4 years; however, the limb salvage rate was only 80% [18].

A previous retrospective multicenter study reported a lower risk of stent occlusion and major target limb amputation after implantation of covered stents measuring 7 or 8 mm in diameter in patients with femoropopliteal lesions receiving DAPT. However, fewer than 15% of patients in that cohort received Viabahn stent grafts ≥ 7 mm [19]. In the present study, nearly half of lesions were treated with Viabahn stent grafts ≥ 7 mm, suggesting that the pave-and-crack technique may facilitate larger-diameter stent graft implantation, with potential long-term clinical benefit, as observed in this study.

Overall, conventional treatment options were limited in the present cohort, as severe calcification and long CTOs often rendered standard endovascular modalities unsuitable. Although previous studies have reported EVT outcomes in complex calcified lesions, defined as PACSS grade 3–4 [20, 21], the PACSS primarily assesses the circumferential distribution and longitudinal extent of calcification, without accounting for plaque density or thickness [22]. In the present study, atherectomy was considered unfeasible and PTA at high risk for vessel rupture. Intravascular lithotripsy (IVL) may represent an alternative treatment strategy. The randomized Disrupt PAD III trial demonstrated promising mid-term efficacy of IVL compared with standard PTA in moderately to severely calcified femoropopliteal lesions. However, lesions were substantially less complex than in the present cohort, with shorter mean lesion length (100 mm) and fewer CTOs (26%). The bailout stenting rate was low following IVL; however, it was also below 20% in the PTA arm, suggesting low lesion complexity compared to our cohort [23]. Future data from the prospective CRACK-IT study (NCT06112171) may clarify the role of IVL. In this study, highly complex femoropopliteal lesions will be randomized to pave-and-crack or IVL for lesion preparation and stenting on indication, without the necessity for covered stents.

### Limitations

This study is limited by its retrospective, single-center design, potential selection bias, and the lack of independent core laboratory assessment. Because data were collected retrospectively, information on primary patency was limited. Therefore, freedom from TLR was selected as a more robust and clinically meaningful endpoint for this study design. In addition, follow-up data were limited by the high mortality rate in this complex patient cohort and loss to follow-up.

## Conclusion

The pave-and-crack technique represents a feasible EVT option for patients unsuitable for open surgical revascularization and may constitute the only viable strategy in some highly calcified femoropopliteal CTOs. In these otherwise difficult-to-treat lesions, the technique was associated with high technical success and low residual stenosis. Long-term clinical outcomes were encouraging, with favorable freedom from TLR and a high limb salvage rate.

## Supplementary Information

Supplementary Material 1.

## Data Availability

The data that support the findings of this study are available on request from the corresponding author, AS. The data are not publicly available to protect the privacy of research participants.

## Abbreviations

CLTI: Chronic limb-threatening ischemia
CTO: Chronic total occlusion
DCB: Drug-coated balloon
DES: Drug-eluting stent
EVT: Endovascular therapy
KM: Kaplan–Meier
PACSS: Peripheral Arterial Calcium Scoring System
PAD: Peripheral artery disease
PTA: Percutaneous transluminal angioplasty
TLR: Target lesion revascularization
