# Supplementary material for: Long-term outcomes of the “pave-and-crack” technique for severely calcified femoropopliteal chronic total occlusions
Source: CVIR Endovasc. 2026 Sep 4;9:112. doi: 10.1186/s42155-026-00766-9 (PMC13545202; doi:10.1186/s42155-026-00766-9)
Supplement: Supplementary file 1 [file 42155_2026_766_MOESM1_ESM.docx]

**Supplements**

**Figure S1.** Kaplan Meier estimates for freedom from target lesion revascularization over five years with corresponding numbers at risk. KM: Kaplan-Meier, SD: Standard deviation, TLR: Target lesion revascularization.

**Figure S2.** Kaplan-Meier analysis for overall survival stratified for clinical presentation at baseline over five years with corresponding risk table. KM: Kaplan-Meier, SD: Standard deviation, CLTI: Chronic limb threatening ischemia.

**Table S1.** Causes of death through 365 days of follow up.

| **Causes of death** | **Number of patients** |
| --- | --- |
| Malignancy | 4 |
| Sepsis/pneumonia | 5 |
| Heart insufficiency | 1 |
| Unknown | 4 |
